# Investigation on the Factors Associated with the Persistence of Anosmia and Ageusia in Saudi COVID-19 Patients

**DOI:** 10.3390/ijerph19031047

**Published:** 2022-01-18

**Authors:** Saad N. Algahtani, Abdullah F. Alzarroug, Hatan K. Alghamdi, Haif K. Algahtani, Nasser B. Alsywina, Khalid A. Bin Abdulrahman

**Affiliations:** College of Medicine, Imam Mohammad Ibn Saud Islamic University, Othman Bin Affan Road Al-Nada, P.O. Box 7544, Riyadh 13317-4233, Saudi Arabia; afmalzarroug@sm.imamu.edu.sa (A.F.A.); Hkialghamdi@sm.imamu.edu.sa (H.K.A.); hksalgahtani@sm.imamu.edu.sa (H.K.A.); nbnalsywina@sm.imamu.edu.sa (N.B.A.); kab@imamu.edu.sa (K.A.B.A.)

**Keywords:** SARS-CoV-2, post-acute COVID-19 syndrome, anosmia, ageusia, COVID-19, Saudi Arabia

## Abstract

The Coronavirus Disease 2019 (COVID-19) outbreak caused by the severe acute respiratory syndrome coronavirus-2 (SARS-CoV-2) resulted in a worldwide pandemic of a highly infectious disease. The difficulty of dealing with COVID-19 is the broad spectrum of clinical manifestations that involves various pathophysiological mechanisms, severities, duration, and complications. This study aims to help emphasize the factors related to the persistence and duration of anosmia (loss of smell) and ageusia (loss of taste) as part of post-acute COVID-19 syndrome in Saudi COVID-19 patients via a retrospective cross-sectional design. Eight hundred and eighty-one participants were recruited between March and April 2021. Those participants were 18 years or older, recovered from the COVID-19 infection, and completed 14 days after the onset of the acute phase of the disease. Among the 881 recruited participants, 808 have submitted eligible responses and were included in data analyses. The most common persistent symptoms in post-acute COVID-19 syndrome were anosmia (33.8%) and ageusia (26.4%). The data also showed a significant association between female sex and the incidence and the persistence of anosmia and ageusia. In multivariable analysis, anosmia during the acute phase was associated with BMI, asthma and shortness of breath, while anosmia during the post-acute phase was associated with sex. Ageusia during the acute phase was associated with sex, myalgia and arthralgia, while ageusia in the post-acute phase was associated with sex.

## 1. Introduction

The Coronavirus Disease 2019 (COVID-19) outbreak caused by severe acute respiratory syndrome coronavirus-2 (SARS-CoV-2) resulted in a worldwide pandemic of a highly infectious disease. The origin of SARS-CoV-2 has been traced back to Wuhan, China, giving rise to an epidemic, eventually causing a global pandemic [1]. As of August 2021, there have been over 200 million confirmed cases of COVID-19, including over 4 million deaths worldwide [2]. The real difficulty in dealing with COVID-19 is the variation of symptoms, severity, duration and complications.

Among the most frequent symptoms reported in patients with COVID-19 are those chemosensitive in nature, such as anosmia (loss of smell) and ageusia (loss of taste). In some instances, these symptoms surpass the duration of the disease and persist long after the disease has subsided [3]. It has been potentially described that the cause of anosmia is not neurological in nature, although it is considered a neurological symptom. It was noticed that the sensory neurons that detect and transmit the smell to the brain are not vulnerable to SARS-CoV-2; instead, the gene is expressed at the epithelium of the nasal cavity. Accordingly, it has been suggested that this is the leading cause of anosmia in COVID-19 patients [4]. Further studies are being conducted to understand the exact pathogenesis of these chemosensitive disorders, yet no studies have yet clarified these symptoms [5]. Regarding ageusia symptoms, it has been suggested that the same gene expressed by the epithelium of the nasal cavity as a cellular receptor for SARS-CoV-2 is diffusely expressed on the mucous membrane of the oral cavity, particularly on the tongue. Since the chemosensitive effect of SARS-CoV-2 is bound to the sialic acid receptor, a component of the salivary mucin that protects the glycoproteins that convey gustatory molecules in the taste pores, it will lead to an increase in the gustatory threshold, thereby causing ageusia [6].

Post-acute COVID-19 syndrome is defined as the persistence of COVID-19 symptoms after four weeks from the onset of the acute phase symptoms [7]. This syndrome involves a broad range of residual symptoms of long COVID or persistent post-COVID syndrome (PPCS), multiorgan consequences of COVID-19, and long-term effects of COVID-19 treatment/hospitalization. The Centers for Disease Control and Prevention (CDC) and other publications in the literature identified anosmia and ageusia among the most common symptoms to be endured during post-acute COVID-19 syndrome [8,9].

The medical field is constantly improving regarding the complications of COVID-19. Although the persistence of anosmia and ageusia has not been fully understood, the encouragement of further studies to search among these symptoms will enhance the clinical approach towards dealing with such complications. This study aims to investigate the factors related to the persistence and duration of anosmia (loss of smell) and ageusia (loss of taste) in Saudi COVID-19 patients.

## 2. Materials and Methods

### 2.1. Study Design

This study adopted a retrospective cross-sectional design via an online distributed self-administered questionnaire. A total of 881 participants who were 18 years or older, recovered from the COVID-19 infection, and completed 14 days after the onset of the initial infection were recruited between the fourth of March 2021 and the twelfth of April 2021. However, individuals who were not infected with COVID-19, individuals currently infected with COVID-19, and patients who were infected before the second of March 2020 were excluded. The distributed questionnaires included 33 questions divided into four sections. All questions were reviewed by an independent ENT consultant, translated from Arabic to English then peer-reviewed for proofreading. Questions and answers were available in both Arabic and English languages. Additionally, participants received the authors’ contact information if they needed assistance filling out the questionnaire.

The first section is mainly concerned with securing participants consent to participate and checking for eligibility to enroll in the study by asking about their clinical diagnosis at the time of enrollment and the exact date of the diagnosis; these variables would be used later to calculate the period of follow-up, which is the time interval between the onset of the infection and the date of the participation in the study. In the second section, participants were asked about their demographical and personal characteristics. Then, in the third section, participants were questioned about their medical history and previous illnesses. Such medical information is essential to determine whether these characteristics could influence the study outcomes.

The last section includes questions about COVID-19 symptoms during and after the infection, their severity, persistence, and associated circumstances (hospitalization, medications, etc.) with a major focus on anosmia and ageusia (onset, quality, provoking factors, time course, etc.).

### 2.2. Ethical Considerations

This study was approved by the Institutional Review Board (IRB) of Imam Mohammad Ibn Saud Islamic University (Project number: 50-2021) and followed the Declaration of Helsinki. Before answering the questionnaire, each participant had signed a consent form to declare their willingness to participate in the study and fully understood the research objectives. Furthermore, all participants were informed that the anonymity of their identities is guaranteed, and their responses will be used for research purposes only.

### 2.3. Sample Size Justification

To justify the number of patients, the prevalence of the condition of interest in the population was assumed to be 10%. For power 90%, type I error rate 5%, and to detect an odds ratio of 2, the following formula was used:

$$n=\frac{{\left(1+r\right)}^{2}{\left(Z_{\frac{\alpha }{2}}+Z_{1-\beta }\right)}^{2}}{r{\left(\ln OR\right)}^{2}[p\left(1-p\right)]}$$

*n =* 4(1.64 + 1.64)^2^/(Ln(2) × (0.1 × 0.9) = 700.

Accordingly, the sample size is then 700 to account for the possible missing data, and 881 subjects were included in the study [10].

### 2.4. Data Analyses

Data were stored in an Excel datasheet, and upon completion of the data collection, it was exported to the SPSS (IBM) version 27. Then, data were analyzed in two steps. The first was to produce descriptive measures of the demographics and the factors of interest via a one-dimensional frequency table or higher dimensional cross-classification tables. The association between the variables was evaluated using the Chi-square test of independence in the second step. The two-by-two tables evaluated the association quantitatively using the Mantel–Haenszel odds ratio (OR) [11]. Values of odds ratio above one mean that the exposure is a risk factor. Values of odds ratios lower than one mean that the exposure has a protective effect on the outcome. A multiple logistic regression analysis was conducted to independently investigate the factors associated with the outcomes. A stepwise procedure was used to fit a logistic regression model. The statistical significance for all analyses was set at *p* < 0.05.

## 3. Results

### 3.1. Demographical and Clinical Features of the Participants

Among the 881 recruited participants, 808 (91.7%) had completed and submitted all mandatory questions, and the remaining 73 (8.3%) patients were excluded due to incomplete responses. About half of the participants were aged 18 to 29 years (*n* = 429, 53.1%) and were females (*n =* 445, 55.1%). The majority of patients were residents of Riyadh region (30.2%), Makkah region (23.1%), Al-Jouf region (16.6%), and Al-Madinah region (15.5%). The mean BMI (SD) was 26.64 (6.4) kg/m^2^. Nearly one-third were overweight (31.1%), and 25.1% were obese. Nearly 50% (*n =* 403) of the participants were unmarried, while 45.2% (*n =* 365) were married. A substantial proportion of the participants were either students (*n =* 297, 36.8%) or working in the government sector (*n =* 216, 26.7%). Intestinal diseases were the most prevalent group of comorbidities; 192 (23.8%) participants reported a history of inflammatory bowel diseases or irritable bowel syndrome. Several other chronic diseases such as diabetes, asthma, and hypertension were reported in 8.3%, 7.8%, and 6.4% of the participants, respectively. Two hundred and sixteen (26.7%) patients have experienced decreased or complete loss of smell and taste at least once before their infection with COVID-19. Smoking cigarettes, hookah, and electronic cigarettes were practiced by 152 (18.8%) participants, along with 72 (8.9%) previous smokers. An aggregate of 538 (66.6%) participants had an infection with seasonal influenza within a year prior to their COVID-19 infection. However, only 147 (18.2%) patients with the influenza virus received seasonal influenza virus vaccine. Past medical and surgical history for ear, nose, or throat complaints were found in 10.6% and 12.13% of the patients, respectively. Most of the participants endured the COVID-19 infection for 14 days or less (*n =* 585, 72%). However, 7.6% (*n =* 65) of them were isolated for more than 22 days after the first positive swab. Most of the participants were isolated in their houses (*n =* 746, 92.3%), 37 (4.6%) needed to be hospitalized, and merely 2 patients needed to be admitted to the intensive care unit (Table 1).

About 167 (20.7%) patients admitted the use of treatments for COVID-19 infection. The most frequent treatments or supplements used were paracetamol (10.6%), vitamin C (8.7%), zinc (7.8%), and antibiotics (not specified) (2.8%). About 184 (34.8%) reported performing physical activities during the isolation period (during the acute phase). The most common signs and symptoms at the onset of illness were anosmia (72%), ageusia (64.2%), headache (59.8%), fever (54.3%), myalgia, and arthralgia (51.5%), and fatigue (46.7%). By the end of the first two weeks after the onset of symptoms, 339 (42%) patients were free of symptoms. The rest of the patients reported several persistent symptoms, which lasted more than 14 days after the onset of symptoms; the most frequent were anosmia (33.8%), ageusia (26.4%), myalgia and arthralgia (13.1%), fatigue (10.8%), dyspnea (9.2%), headache (9%), and cough (7.4%). Anosmia and/or ageusia had, in general, longer-term persistence in comparison to other COVID-19 symptoms, 23.9% of the patients who suffered from loss of taste and/or smell suffered from persistent anosmia and/or ageusia for 22 days or more after the end of the acute phase in comparison to 17.3% of the patients who suffered from other COVID-19 symptoms (Table 2).

### 3.2. Factors Associated with Incidence and Persistence of Anosmia and Ageusia

**Figure 1:**
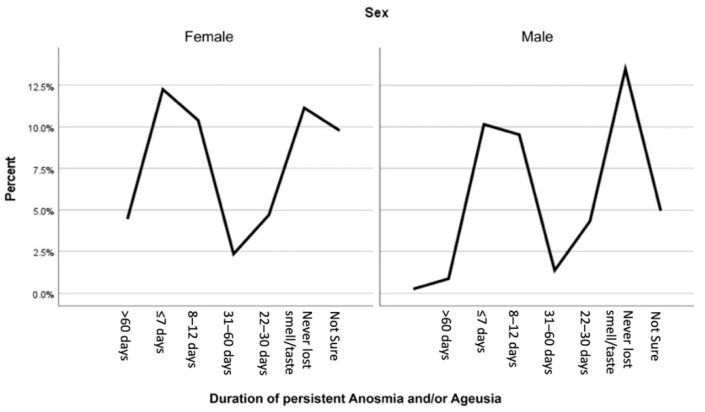
The association between sex and the duration of persistent anosmia and/or ageusia (*p* = 0.001).

There was a significant association between female sex with both the incidence and the persistence of anosmia and ageusia. Females were more prone to endure anosmia during the infection in comparison to males (58.6% vs. 41.4%) (*p* = 0.001) and after the end of the acute phase (67% vs. 33%) (*p* = 0.001) (Table 3). On the other side, females were also found to be more susceptible to experience ageusia during the infection in comparison to males (59.2% vs. 40.8%) (*p* = 0. 002), and this also applies to persistent ageusia (69.5% vs. 30.5%) (*p* = 0. 0001) (Table 4). Moreover, females tend to be affected by both anosmia and ageusia for more extended periods of time in comparison to males; this is evident by the fact that 83.7% of the patients who had anosmia and/or ageusia that persisted for more than 60 days were females (*p* = 0.001) (Figure 1).

A high incidence of anosmia during COVID-19 was found in younger patients, especially those in the third (44%) and fourth (22.2%) decades age group in comparison to older age groups (*p* = 0.001). In addition, those patients in the third and fourth decades were also associated to a lower extent with the occurrence of persistent anosmia by (43.2%) and (41.9%), respectively (*p* = 0.016) (Table 3). Regarding ageusia, age has a significant association with experiencing ageusia as one of the initial symptoms of COVID-19. Many of the participants who presented with ageusia as an initial symptom of COVID-19 infection were from the third (42.6%) and fourth (23.3%) decades age group, establishing a significant association between age and loss of smell during the infection (Table 4). However, there was no significant association between age and the persistence of ageusia.

**Figure 2:**
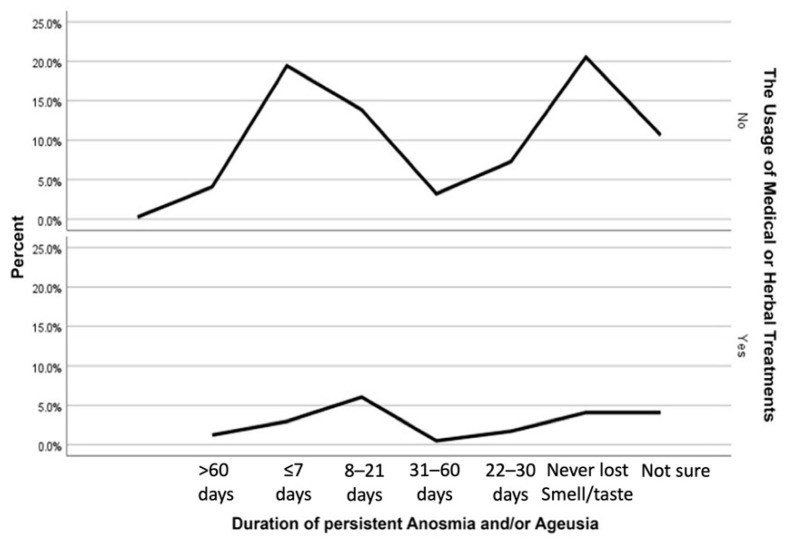
The association between the use of treatments and the duration of persistent anosmia and/or ageusia (*p* = 0.002).

The odds of using treatments (targeting COVID-19) during the course of the infection is increased by 50% in individuals who had persistent anosmia (*p* = 0.021) (Table 3), 61% in the patients who presented with ageusia during the infection (*p* = 0.013), 85% in the subjects who presented with persistent ageusia in comparison to those who did not (*p* = 0.001) (Table 4). Though, no significant association was found between loss of smell during the infection and the use of treatments. Interestingly, the use of treatments also showed a significant association with the duration of persistent anosmia and/or ageusia (Figure 2).

Furthermore, the likelihood of having a previous medical history of intestinal diseases is 62% higher in subjects who experienced persistent anosmia. In addition, several medical conditions were significantly associated with the persistence of anosmias, such as liver diseases (*p* = 0.020) and cancer (*p* = 0.028). All other studied factors were found insignificantly associated with the incidence and persistence of anosmia and ageusia.

### 3.3. Symptoms Associated with Anosmia and Ageusia

This study also shows that loss of smell during COVID-19 infection was associated with several other COVID-19 symptoms, including cough, chest pain, runny or congested nose, sore throat, diarrhea, constipation, nausea, stomachache, fever, loss of appetite, insomnia, fatigue, headache, and myalgia and arthralgia. On the other hand, the persistence of anosmia was associated with hearing difficulties, chest pain, and headache.

Several other symptoms were identified to be associated with loss of taste during the infection, including hearing difficulties, chest pain, runny or congested nose, sore throat, diarrhea, rash, nausea, stomachache, fever, loss of appetite, insomnia, fatigue, headache, myalgia, and arthralgia. Persistent ageusia was associated with dyspnea, persistent anosmia, hearing difficulties, chest pain, and headache (Table 5).

### 3.4. Characteristics of Anosmia and Ageusia

**Figure 3:**
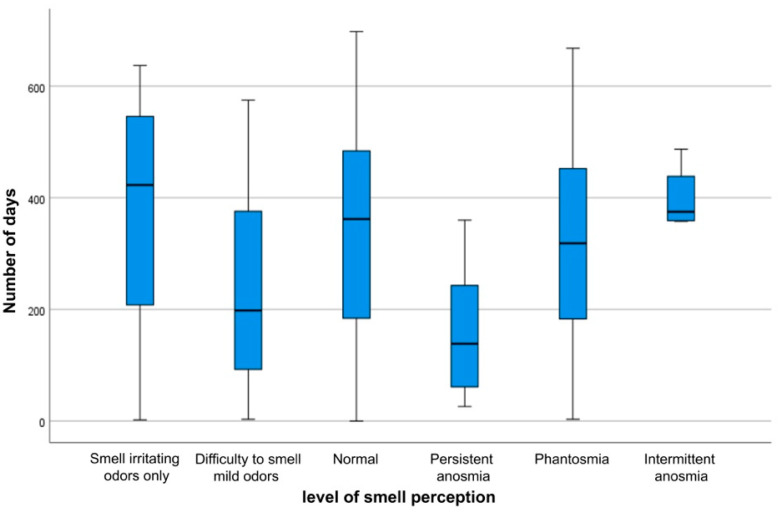
Boxplot of the number of days for each stage of loss of smell.

**Figure 4:**
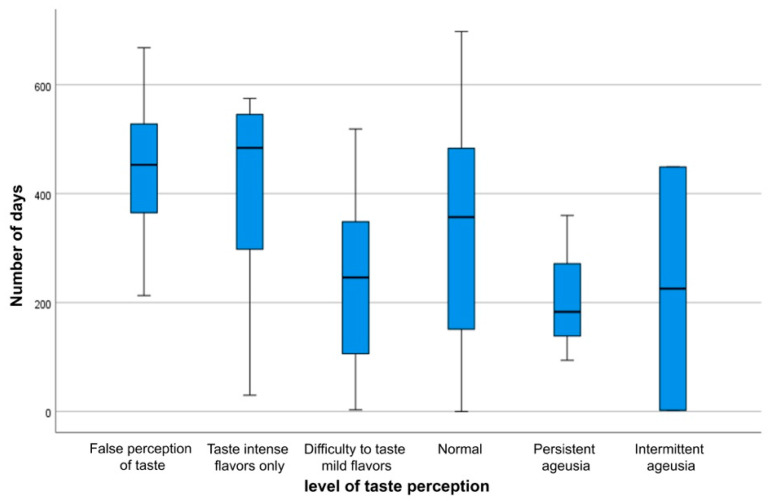
Boxplot of the number of days for each stage of loss of taste.

The healing process and the characteristics of loss of smell and taste are not solely influenced by personal and clinical factors, but time also has an impact on the duration of these two symptoms. As the time interval between the onset of the infection and the date of the participation in the study (period of follow-up) changes, the status of smell and taste varies, which rejects the hypothesis of a constant median number of days of follow-up periods across different stages of loss of smell (Figure 3) (*p* = 0.0001) and loss of taste (Figure 4) (*p* = 0.0001). This indicates that the number of follow-up days is significantly associated with the features of subsiding anosmia and ageusia.

### 3.5. Factors Associated with Anosmia and Ageusia in Multivariable Analysis

In multivariable analysis, anosmia during the acute phase was associated with BMI, asthma and shortness of breath, while anosmia during the post-acute phase was associated with sex. Ageusia during the acute phase was associated with sex, myalgia, and arthralgia, while ageusia in the post-acute phase was associated with sex (Table 6).

## 4. Discussion

Our study was a survey-based investigation involving 808 COVID-19 patients. The main objectives of the study were two-fold, firstly; to quantify the incidence and prevalence of anosmia and ageusia in COVID-19 patients; second, to identify the antecedent risk factors associated with both conditions. We showed that both conditions were highly prevalent among the surveyed patients: the prevalence of anosmia was 72%, and 64.2% for ageusia. Our results were in close agreement with results reported by Lechien et al. (2020) [3]. In their report, the most common symptom in COVID-19 patients was olfactory in nature (85.6%), of whom 79.6% were anosmic, and 20.4% were hyposmic. However, the persistence of olfactory dysfunction was 63%, while in our study, it was 33.8%. This variation in levels of persistence signifies the importance of understanding these symptoms and their associated factors.

The most significant factor associated with the occurrence and persistence of anosmia and ageusia was gender. Anosmia and ageusia persistence in females were 67% and 69.5%, respectively, in comparison to males (33%) and (30.5%), respectively. A study conducted by Lee et al. (2020) [12] showed that females were more prone to anosmia and ageusia (68.9%) in comparison to males (31.1%). Another research showed that anosmia was more prevalent among females [13]. These findings may emphasize the importance of investigating gender differences of immune responses towards COVID-19.

Anosmia was also associated with age during infection, where patients in the third (44%) and fourth (22.2%) decades were significantly affected. This association was also reported in a previous study [12]. They showed that younger individuals were more susceptible to anosmia and ageusia. However, regarding persistence, no association was found. This indicates that younger patients are more susceptible to the infection.

One of the symptoms associated with anosmia and ageusia during infection was the runny or congested nose. However, it did not show any relevant association with the persistence of anosmia and ageusia. Thus, it presented in COVID-19 patients as an isolated symptom, or it may have hindered their perception of taste and smell, which led them to report anosmia and ageusia as a symptom. Anosmia and ageusia progression to regaining these senses is associated with time; however, the relation is not constant, and regaining taste and smell is subjective to every patient. Alternately, the level of severity of COVID-19 was not associated with anosmia and ageusia, further emphasizing the subjectivity of progression. Therefore, further studies need to be carried out to understand the nature of the associations. This study found a significant association between anosmia and headache. Recent findings have shown that anosmia is associated with neurological dysregulations. Indeed, the common main symptoms attributable to central nervous system involvement include headache, anosmia, and dysgeusia [14].

This study found a significant association between body mass index and anosmia in multivariable analysis. In the literature, there is no direct report available on the loss of these chemical senses in obese COVID-19 patients. However, the association between obesity and impairment of the oro-naso-sensory perception was reported in the literature [15]. Krams et al. (2020) suggested that the obesity paradox provides a possible explanation for the observed population differences in COVID-19 infection and mortality rates between Europe, the USA, and Southeast Asia. In the obesity paradox, obese patients may have better health outcomes than normal-weight patients despite the greater risk of local and systemic inflammation in their fat tissue [16]. It is well-known that ACE2 is highly expressed in adipose tissue, especially in visceral fat, suggesting an essential role for this tissue in determining COVID-19 disease severity and duration [14,17].

It is important to report that in the early stages of the pandemic, ageusia and anosmia were not regarded as a symptom indicated for screening cases by international bodies. The frequency of these symptoms and their association were only made possible for detection by clinical experience [18,19].

Limitations of the study include that the number of participants in different age groups was not similar. More participants in the elderly age group are needed for further relations. The questionnaire did not include a question separating the course of recovery of ageusia and anosmia as independent symptoms. A timeline could not be established because the survey questions were closed-ended. More participants are needed in the groups of chronic diseases, such as hypertension and diabetes, for further association. The questionnaire was self-administered, which might induce self-reporting bias and recall bias. Among the symptoms that can appear in SARS-CoV-2 infection that the authors did not consider is dizziness, which can be a symptom of onset [20].

## 5. Conclusions

In our findings, the most prevalent symptoms during the acute phase of COVID-19 infection were anosmia and ageusia. Furthermore, the most common persistent symptoms were anosmia and ageusia. In addition, a significant association between female sex with both the incidence and the persistence of anosmia and ageusia was established. Hopefully, these findings will enrich our understanding of the nature of those two symptoms and encourage further studies to enhance the knowledge toward post-acute COVID-19 syndrome.

## Figures and Tables

**Table 1:** Clinical and demographical features of COVID-19 patients.

1. Demographical Features	*n* (%)	2. Clinical Features	*n* (%)
1.1 Sex		2.1 Medical History (Prior to COVID-19 infection)	
Female	445 (55.1)	Diabetes	67 (8.3)
Male	363 (44.9)	Hypertension	52 (6.4)
1.2 Age		Cardiac diseases	15 (1.9)
18–19 years	87 (10.8)	Asthma	63 (7.8)
20–29 years	342 (42.3)	Pulmonary diseases	36 (4.5)
30–39 years	174 (21.5)	Hepatic diseases	6 (0.7)
40–49 years	127 (15.7)	Intestinal diseases	192 (23.8)
50–59 years	58 (7.2)	Cancer	5 (0.6)
60–69 years	16 (2)	Loss of taste and/or smell	216 (26.7)
≥70 years	4 (0.5)	Smokers	152 (18.8)
1.3 Body Characteristics		Previous smokers	72 (8.9)
BMI * (kg/m^2^)	26.64 ± 6.4	2.2 Influenza virus infection (last year)	
-Underweight	40 (4.95)	None	263 (32.5)
-Normal weight	314 (38.86)	Once	327 (40.5)
-Overweight	251 (31.06)	Twice	145 (17.9)
-Obese	203 (25.12)	Three times	40 (5)
1.4 City of residence		>Three times	26 (3.2)
Riyadh Region	244 (30.2)	Not sure	7 (0.87)
Makkah Region	187 (23.1)	2.3 Influenza virus vaccination (last year)	147 (18.2)
Al-Jouf Region	134 (16.6)	2.4 ENT Surgeries (last year)	95 (12.13)
Madinah Region	125 (15.5)	2.5 ENT Complaints (last year)	87 (10.6)
Eastern Region	29 (3.6)	Sinusitis	18 (2.23)
Al-Qassim Region	19 (2.4)	Allergic rhinitis	8 (1)
Najran Region	17 (2.1)	2.6 Duration of COVID-19 infection	
Ha’il Region	16 (2)	≤7 days	148 (18.3)
Asser Region	14 (1.7)	8–14 days	437 (54.1)
Other Regions	22 (2.8)	15–21 days	104 (12.9)
1.5 Marital status		22–30 days	45 (5.6)
Single	403 (49.9)	31–60 days	12 (1.5)
Married	365 (45.2)	≥61 days	8 (0.5)
Divorced	28 (3.5)	Not sure	54 (6.7)
Widowed	12 (1.5)	2.7 Isolation site	
1.6 Employment Status		Home isolation	746 (92.3)
Student	297 (36.8)	Hospital Admission	37 (4.6)
Government employee	216 (26.7)	ICU admission	2 (0.2)
Unemployed	155 (19.2)	Government isolation facility	23 (2.8)
Private sector employee	97 (12)		
Freelancer	43 (5.3)		

* BMI classification is based on the National Heart, Lung, and Blood Institute categorization (underweight ≤ 18.5, normal weight = 18.5–24.9, overweight = 25–29.9, obesity = BMI ≥ 30).

**Table 2:** Clinical features of COVID-19 patients during the acute phase of the infection.

2. Clinical Features	*n* (%)	2. Clinical Features	*n* (%)
2.8 Physical activity during isolation period	282 (34.9)	2.13 Persistent symptoms	
2.9 Medications for COVID-19	167 (20.7)	Asymptomatic	339 (42)
Paracetamol	86 (10.6)	Anosmia	273 (33.8)
Vitamin C	70 (8.7)	Ageusia	213 (26.4)
Zinc	63 (7.8)	Myalgia and arthralgia	106 (13.1)
Unspecified antibiotic	23 (2.8)	Fatigue	87 (10.8)
Azithromycin	14 (1.7)	Dyspnea	74 (9.2)
Vitamin D	13 (1.6)	Headache	73 (9)
Multi-vitamins	11 (1.4)	Cough	60 (7.4)
Unspecified antipyretic	9 (1.1)	Insomnia	47 (5.8)
Other	37 (4.5)	Chest pain	41 (5.1)
2.10 Self-rated severity of the symptoms		Runny or congested nose	32 (4.0)
Mild	222 (27.5)	Loss of appetite	30 (3.7)
Moderate	389 (48.1)	Diarrhea	24 (3)
Severe	159 (19.7)	Sore throat	23 (2.8)
Asymptomatic	38 (4.7)	Nausea	18 (2.2)
2.11 Acute-phase symptoms		Hearing difficulties	14 (1.7)
Anosmia	582 (72)	Visual disturbance	14 (1.7)
Ageusia	519 (64.2)	Stomachache	9 (1.1)
Headache	483 (59.8)	Constipation	7 (0.9)
Fever	439 (54.3)	Rash	6 (0.7)
Myalgia and arthralgia	416 (51.5)	Fever	5 (0.6)
Fatigue	377 (46.7)	2.14 Current level of smell perception	
Cough	309 (38.2)	Normal perception of smell	378 (46.8)
Runny or congested nose	305 (37.7)	Difficulty smelling mild odors	70 (8.7)
Dyspnea	278 (34.4)	smell irritating odors only	49 (6.1)
Loss of appetite	274 (33.9)	Phantosmia	45 (5.6)
Sore throat	244 (30.2)	Intermittent anosmia	25 (3.1)
Insomnia	198 (24.5)	Persistent anosmia	18 (2.2)
Chest pain	179 (22.2)	Never lost smell **	217 (26.9)
Diarrhea	156 (19.3)	Not sure	6 (0.7)
Nausea	148 (18.3)	2.15 Current level of taste perception	
Stomachache	72 (8.9)	Normal perception of taste	388 (48)
Asymptomatic	45 (5.6)	Difficulty to taste mild flavors	59 (7.3)
Hearing difficulties	43 (4.2)	taste intense flavors only	34 (4.2)
Visual disturbance	37 (4.6)	False perception of taste	19 (2.4)
Constipation	31 (3.8)	Intermittent ageusia	16 (2)
Rash	18 (2.2)	Persistent ageusia	8 (1)
2.12 Time is taken for symptoms to subside (other than ageusia and anosmia) (*n =* 770) *		Never lost taste **	278 (34.4)
		Not sure	6 (0.7)
≤7 days	247 (32)	2.16 Time taken for Ageusia and/or Anosmia to subside (*n =* 607) ***	
8–21 days	260 (33.8)		
22–30 days	67 (8.7)	≤7 days	181 (29.8)
30–60 days	35 (4.5)	8–21 days	161 (26.5)
>60 days	31 (4)	22–30 days	73 (12)
Not sure	130 (16.9)	30–60 days	30 (4.9)
		>60 days	43 (7)
		Not sure	119 (19.6)

* 38 patients out of the 808 were asymptomatic during and after the infection and were not asked to answer this question. ** Participants who did not experience ageusia or anosmia during and after their infection. *** 201 patients out of the 808 have not experienced both anosmia and ageusia during and after the infection and were not asked to answer this question.

**Table 3:** Demographic and clinical factors associated with experiencing anosmia.

Variables		Presence of Anosmia during the Infection *n* (%)	Absence of Anosmia during the Infection *n* (%)	OR	*p*
(3.1) During COVID-19 acute phase:					
A. Sex	Male	241 (41.4)	122 (54)	0.60	0.001
	Female	341 (58.6)	104 (46)		
B. Age	18–19	58 (10)	29 (12.8)	-	0.001
	20–29	256 (44)	86 (38.1)		
	30–39	129 (22.2)	45 (19.9)		
	40–49	96 (16.5)	31 (13.7)		
	50–59	36 (6.2)	22 (9.7)		
	60–69	7 (1.2)	9 (4)		
	≥70	0	4 (1.8)		
(3.2) As a persistent symptom (post-acute phase):					
A. Sex	Male	90 (33)	273 (51)	0.47	0.001
	Female	183 (67)	262 (49)		
B. Age	18–19	24 (8.8)	63 (11.8)	-	0. 016
	20–29	118 (43.2)	224 (41.9)		
	30–39	64 (23.4)	110 (20.6)		
	40–49	53 (19.4)	74 (13.8)		
	50–59	12 (4.4)	46 (8.6)		
	60–69	2 (0.7)	14 (2.6)		
	≥70	0	4 (0.7)		
C. Liver diseases	Yes	4 (1.5)	2 (0.4)	4.02	0.020
	No	265 (97.1)	532 (99.4)		
D. Intestinal diseases	Yes	81 (29.7)	111 (20.7)	1.62	0.018
	No	179 (65.6)	398 (74.4)		
E. Cancer	Yes	4 (1.5)	1 (0.2)	7.94	0.028
	No	269 (98.5)	534 (99.8)		
F. Medications	Yes	69 (25.3)	98 (18.3)	1.5	0.021
	No	204 (74.7)	437 (88.4)		

**Table 4:** Characteristics of the participants in association with experiencing ageusia.

Variables		Presence of Ageusia during the Infection *n* (%)	Absence of Ageusia during the Infection *n* (%)	OR	*p*
(4.1) During COVID-19 acute phase:					
A. Sex	Male	212 (40.8)	151 (52.2)	0.63	0. 002
	Female	307 (59.2)	138 (47.8)		
B. Age	18–19	54 (10.4)	33 (11.4)	-	0.031
	20–29	221 (42.6)	121 (41.9)		
	30–39	121 (23.3)	53 (18.3)		
	40–49	83 (16)	44 (15.2)		
	50- 59	33 (6.4)	25 (8.7)		
	60–69	7 (1.3)	9 (3.1)		
	≥70	0	4 (1.4)		
C. Medications	Yes	121 (23.3)	46 (15.9)	1.61	0.013
	No	398 (76.7)	243 (84.1)		
(4.2) As a persistent symptom (post-acute phase):					
A. Sex:	Male	65 (30.5)	298 (50.1)	0.44	0. 0001
	Female	148 (69.5)	297 (49.9)		
B. Duration of COVID (By days)	≤7	43 (20.2)	105 (17.6)	-	0.001
	8–14	121 (56.8)	316 (53.1)		
	15–21	40 (18.8)	64 (10.8)		
	30	5 (2.3)	40 (6.7)		
	60	4 (1.9)	8 (1.3)		
	≥90	0	8 (1.3)		
	Not sure	0	54 (9.1)		
C. Medications	Yes	61 (28.6)	106 (17.8)	1.85	0.001
	No	152 (71.4)	489 (82.2)		

**Table 5:** List of the associated symptoms with anosmia and ageusia.

**Symptoms Associated with Anosmia during the Acute Phase**			**Symptoms Associated with Ageusia during the Acute Phase**		
**Name of the Symptoms**	**OR**	***p***	**Name of the Symptoms**	**OR**	***p***
A. Cough	1.47	0.02	A. Hearing difficulties	2.211	0.059
B. Chest Pain	1.79	0.004	B. Chest pain	1.82	0.001
C. Runny or congested nose	2.01	0.001	C. Runny or congested nose	1.56	0.004
D. Sore throat	1.76	0.002	D. Sore throat	1.622	0.003
E. Diarrhea	1.64	0.021	E. Diarrhea	1.53	0.028
F. Constipation	3.75	0.021	F. Rash	0.69	0.437
G. Nausea	1.58	0.035	G. Nausea	1.636	0.014
H. Stomachache	2.05	0.025	H. Stomachache	2.47	0.002
I. Fever	1.55	0.005	I. Fever	1.68	0.001
J. Loss of appetite	2.06	0.001	J. Loss of appetite	2.53	0.001
K. Insomnia	2.31	0.001	K. Insomnia	2.35	0.001
L. Fatigue	1.95	0.001	L. Fatigue	1.9	0.001
M. Headache	1.7	0.001	M. Headache	1.90	0.001
N. Myalgia and arthralgia	1.65	0.001	N. Myalgia and arthralgia	1.75	0.001
**Symptoms associated with persistent anosmia**			**Symptoms associated with persistent ageusia**		
**Name of the symptoms**	**OR**	***p***	**Name of the symptoms**	**OR**	***p***
A. Hearing difficulties	5.05	0.003	A. Hearing difficulties	3.8	0.008
B. Chest pain	0.459	0.047	B. Chest Pain	0.2	0.005
C. Headache	2.46	0.001	C. Headache	2.7	0.001
			D. Dyspnea	2.3	0.001
			E. Anosmia	-	0.001

**Table 6:** Factors associated with anosmia and ageusia in multivariable analysis.

Variables	Odds Ratio	*p*
(6.1) Anosmia during the acute phase		
A.BMI	1.04	0.038
B. Asthma	3.27	0.027
C. Shortness of breath	2.73	0.001
(6.2) Ageusia during the acute phase		
A. Female sex	1.80	0.024
B. Myalgia and arthralgia	1.89	0.013
(6.3) Persistence of anosmia post-acute phase		
A. Female sex	1.73	0.045
(6.4) Persistence of ageusia post-acute phase		
A. Female sex	2.09	0.014

## Data Availability

All data generated or analyzed during this study are included in this published article and the References section.
